# How Carequality, The Sequoia Project, and eHealth Exchange Support the Interoperable Exchange of Health Data in the USA

**DOI:** 10.1007/s10278-021-00538-y

**Published:** 2022-09-07

**Authors:** Blanca “Didi” Davis, Alan Swenson

**Affiliations:** 1 https://sequoiaproject.org/; 2 https://carequality.org/

**Keywords:** Interoperability, HIE, Standards, IHE, HL7, DICOM

## Abstract

Every organization in the health IT industry plays an important role in overcoming barriers to health information exchange in the United States. It is important to understand imaging interoperability in the overall context of Health Information Exchange (HIE). The rapid evolution of storage, bandwidth and network transport technologies has made the handling of imaging data converge with the primarily text-based healthcare data. The radiology community must understand the overall environment and become a tightly integrated part of it. As the health IT ecosystems continue to evolve, it became clear that there would not be a single health information exchange network to service the nation. Rather, like other industries such as telecom and banking, there would be multiple networks that would need to interconnect. To support compliance to interoperability standards and specifications, The Sequoia Project began collaborating with industry to create testing programs and tooling that supports transport, security and content testing requirements for four production testing programs today. These testing programs validate compliance to standards for transport and security as well standards for the payloads such as clinical documents and imaging data. While once operating under the same umbrella, The Sequoia Project, Carequality and eHealth Exchange (https://ehealthexchange.org/) have been separate companies since 2018. Each plays a unique role in helping patient information move where and when it is needed, each working with a framework of standards published by IHE, DICOM, and HL7 to enable health information exchange.

## Introduction

Interoperability is a multifaceted challenge that requires multidimensional solutions. It is important to understand imaging interoperability in the overall context of Health Information Exchange. Just a few years ago, many thought that image exchange was very different from general Health information exchange. The rapid evolution of storage, bandwidth, and network transport technologies has made the handling of imaging data converge with the primarily text-based healthcare data. The radiology community must understand the overall environment and become a tightly integrated part of it. “Care providers with access to a patient’s imaging history make better informed care decisions and provide more efficient, higher quality care. Patients with access to their medical images and reports enjoy improved communication that enables them to take more control of their medical care,” said Didi Davis, VP, Informatics, Conformance & Interoperability for The Sequoia Project.

Every organization in the health IT industry plays an important role in overcoming barriers to health information exchange in the USA. While once operating under the same umbrella, The Sequoia Project, Carequality, and eHealth Exchange have been separate companies since 2018. Each plays a unique role in helping patient information move where and when it is needed, each working with each other through a framework of standards published by IHE [11], DICOM [5] and HL7 [2].

## The Sequoia Project [3]

The Sequoia Project is an independent, trusted advocate for nationwide health information exchange that for the public good, stewards’ programs, incubates new initiatives, and educates the community on interoperability and more. eHealth Exchange [9] and Carequality [8] were once initiatives of The Sequoia Project, and in December 2018, became their own companies after successfully being launched by The Sequoia Project.

### The History

In 2012, The Sequoia Project assumed stewardship of the nationwide health information network exchange, NwHIN Exchange, from the Office of the National Coordinator for Health Information Technology (ONC), which later was renamed eHealth Exchange. While it was an initiative of The Sequoia Project, the eHealth Exchange network quadrupled in size, connecting participants across the country and supporting over 120 million patients. Recognizing the maturity and sustainability of the network, eHealth Exchange became independent from The Sequoia Project in 2018.

As the health IT ecosystem continued to evolve, it became clear that there would not be a single health information network to service the nation. Rather, like other industries such as telecom and banking, there would be multiple health information networks that would need to interconnect. To address this need, The Sequoia Project convened stakeholders across the private sector and government to develop a framework to interconnect networks, which led to the creation of Carequality. Carequality also had immense success as an initiative, which led to its relaunch as an independent non-profit in 2018.

### Current Initiatives of The Sequoia Project

Today, The Sequoia Project supports three major initiatives and tackles barriers to interoperability, such as *Patient Matching*, improving usability of data, addressing compliance with information blocking regulations, supporting emergency preparedness, and improving conformance to interoperability standards. This work is facilitated through three programs:

- Interoperability Matters
- Sequoia Interoperability Testing
- RSNA Image Share Validation

#### Interoperability Matters

*Interoperability Matters* is a public-private cooperative that is solving discrete health information exchange challenges. It was launched in 2018 and engages experts from across the healthcare and healthcare IT communities to identify, prioritize, and collaborate on the most pressing barriers and challenges to nationwide health information exchange. Members of The Sequoia Project collaborate to solve targeted, high impact interoperability issues through workgroups.

Currently, Interoperability Matters supports three workgroups:

- Information Blocking Compliance Workgroup
  - Consists of three subgroups: Health Care Providers, Health IT Developers, and Health Information Networks (HINs)/Health Information Exchanges (HIEs)
- Data Usability Workgroup
- Emergency Preparedness Information Workgroup

There are over 400 volunteers and 300 organizations participating across all workgroups and subgroups.

Interoperability Matters provides a plan of action for the healthcare sector to leverage and implement in order to minimize, or better yet eliminate, particular barriers to information exchange.

#### Sequoia Interoperability Testing

The Sequoia Project began collaborating with IHE Services in 2017 to improve the IHE Gazelle Platform to support the requirements for Content, Transport and Security Testing Capabilities outlined by four testing programs in production today.

##### Transport and Security Testing 

The Sequoia Project Interoperability Testing Platform (ITP) was developed for a variety of implementers and verifies that the systems used by their clients comply with the IHE IT Infrastructure Technical Framework [1] specifications prior to being implemented in the production environment.

The best-in-class suite of transport and security testing tools are leveraged today by both health information exchange networks and health IT vendors. Built in partnership with IHE International and IHE Services, the testing toolkit includes:

- Gazelle Test Management (User Management)
- Gazelle Security Suite (Security Testing)
- Assertion Manager (Assertion Testing)
- EVS Client (User Interface for Systems Under Test)
- Patient Manager (Patient Matching and other workflow specific testing)
- Gazelle Web Service Tester
- NIST XDS and FHIR Toolkit (Query and Retrieve Testing)

##### Content Testing

Data exchange is just the beginning for successful health data sharing. Sequoia is also committed to ensuring the content of those data exchanges is both accurate and useful to providers and patients. Sequoia launched the content testing program in February 2018 to improve the quality of data exchanged.

Industry-wide pain points to quality and accurate data content include:

- Optionality: More than one way to do things and inconsistent implementation across vendors
- Terminology: Inconsistent terminology usage
- Specification ambiguity: Narrowed and clarified specifications are needed.
- Complexity: The HL7 C-CDA standard is difficult to understand and consume, and is lacking in clearly documented examples.

The content testing tool supports various HL7 CDA Standards (i.e., HITSP C32/CCD, HL7 C-CDA R1.1, and R2.1 as well as the *US Core Data for Interoperability* (USCDI) versions and associated companion guides. The Sequoia Project plans to support incremental improvements to the quality of data exchanged over time to align with future releases of the USCDI and with future industry implementation guidance published by the Data Usability Workgroup and in support of evolving standards.

#### RSNA Image Share Validation Program

The RSNA Image Share Validation program was developed in partnership with the Radiological Society of North America (RSNA) starting in 2016. The program was updated and improved in 2018 when infrastructure and security testing requirements were added to ensure trust is maintained for the data exchanged by implementers. The program components including the testing documentation, test scripts, and tooling were donated to support the IHE International Conformance Assessment Program [4] components for the DICOM [5], IHE XDS-I [6], and XCA-I [7] Profiles in 2018.

Both programs test the compliance of vendor systems using quality standards determined most effective for accurate and efficient exchange of medical images. With effective standards for image sharing already well established, a validation program is the logical next step. The testing program benefits patients and providers with improved efficiency, reduced costs, enhanced quality of care, and standards-based interoperability to support innovation.

Vendor products that successfully pass a rigorous set of required conformance tests receive the RSNA Image Share Validation seal to communicate to current and future customers their image sharing capabilities. In addition, there is a reciprocity arrangement between the IHE Authorized Conformity Assessment Labs and the Sequoia Project allowing vendors to test once and achieve validation seals for both a US and International conformance.

Care providers with access to a patient’s imaging history make better informed care decisions and provide more efficient, higher quality care. Patients with access to their medical images and reports enjoy improved communication that enables them to take more control of their medical care.

## Carequality [8]

Imagine you had a cell phone plan that only allowed you to call other customers within the same carrier network. That is the situation most healthcare providers experience when joining a data sharing network. Carequality is a network-to-network trust framework that brings together the entire healthcare industry to overcome this challenge by providing a national-level, consensus-built, common interoperability framework to enable exchange between and among health data sharing networks. Representatives from electronic health record (EHR) vendors, record location service (RLS) providers, Health Information Exchanges (HIEs), and other networks from the private and government sectors, come together to determine the technical and policy agreements to enable data to flow between and among the various networks and platforms nationwide.

Carequality implements three essential elements as the core of its operational framework for connecting the country through existing elements:

### Common Rules of the Road

In order for varied implementers of the framework to trust each other with health information, everyone needs to have the same legal obligation to abide by the same rules. Carequality maintains legal and governance documents that remediate the need for having individual business associates or other legal agreements between individual organizations.

### Well-defined Technical Specifications

Shared rules are not enough. Exchange standards must be clearly laid out in an implementation guide that all implementers can easily follow and be held accountable to enable interoperability of the various types of data exchanged.

### A Participant Directory

To connect using the common standards, systems must know the addresses and roles of each participant.

The Carequality Interoperability Frameworks is a comprehensive collection of multiple elements, including legal terms, policy requirement, technical specifications, implementation guides, and more, to provide a clear, detailed and practical approach for health information networks, vendors, and others across the interoperability ecosystem to adopt and ease connectivity.

Carequality supports over 300 million documents exchanged every month and empowers more than 600,000 Physicians, 50,000 clinics and over 4200 hospitals.

RSNA partnered with Carequality in 2019 to jointly develop an implementation guide to address image exchange leveraging the query-based document exchange workflow already in production by multiple networks. The thought was that the image exchange implementation guide would spell out the specific technical requirements to enable exchange of DICOM images in place of CDA Documents, but would leverage the same infrastructure standards used for security and transport published by the Integrating the Healthcare Enterprise (IHE) Radiology Domain. The first draft of the Imaging Supplemental IG was highlighted and published during the SIIM Annual conference on June 26, 2019. The *Proposed — Image Exchange Implementation Guide Supplement v0.2* was open for public comment July–September 2019. Comments were resolved and *next steps were announced* by RSNA and Carequality at a joint *webinar* socializing the updated version of the Draft IG with a call for early adopters to join Carequality in October 2019.

On Monday, December 2, the revised *Carequality Image Exchange Implementation Guide Supplement* [10] was presented at a *town hall* luncheon at the RSNA 2019 Annual Meeting. Before a crowd of representatives from radiology and the medical imaging industry, David S. Mendelson, MD, FACR, Senior Associate, Clinical Informatics and Vice Chair of Radiology IT at The Mount Sinai Health System, and Curtis Langlotz, MD, PhD, Professor of Radiology and Biomedical Informatics Research at Stanford University Medical Center, presented the importance of data liquidity and seamless sharing of medical images by leveraging the existing Carequality Interoperability Framework. At that time, three pioneering imaging vendors (Ambra, Life Image, and Philips) committed to empowering their customers to share imaging studies in 2020 and beyond.

The town hall luncheon closed with a call for the broader imaging community to follow in the footsteps of these pioneers and adopt the Carequality Interoperability Framework. The initial three imaging vendors were joined by two additional implementers (Nuance and Hyland) in 2020 and 2021, respectively. The initial three early adopters began testing in 2020 with each other to verify that the drafted implementation guide was ready to be formally adopted by the Carequality Steering Committee with no required updates. There were some delays due to the COVID-19 pandemic, but the testing did not show any required updates. The draft implementation guide was presented to the Carequality Steering Committee for approval and published for production use in March 2021.

The five image exchange use case implementers will lead the imaging community in making radiology images more accessible to patients and their care teams and eliminating the need to hand-carry or mail disks.

## eHealth Exchange [9]

Active in all 50 states, eHealth Exchange is the largest healthcare information network in the country today, connecting federal agencies and non-federal healthcare organizations so medical data can be exchanged nationwide to improve public health. As a health information network, eHealth Exchange provides healthcare organizations a single on-ramp or connection to exchange patients’ medical data nationwide.

It is difficult for providers to get the medical information they need to provide the appropriate care. eHealth Exchange makes it possible for providers to securely, accurately and electronically exchange health information, improving the care and cost of the patient’s care. Network participants create one connection to eHealth Exchange’s hub and are then connected to shared patient records with all other eHealth Exchange participants.

eHealth Exchange participants mutually agree to support a common set of standards and specifications that enable the establishment of a secure, trusted, and interoperable connection among all organizations participating within the eHealth Exchange network. Participants establish a flow of information by:

- Sending health information to other participating organizations
- Matching patients to their data without a national patient identifier
- Finding and requesting copies of healthcare information from other participating organizations where permitted by law and policy
- Subscribing to receive updates to health information

eHealth Exchange is a Carequality implementer, allowing it to exchange with participants of other Carequality-connected networks. With connectivity that spans all 50 states, eHealth Exchange connects five federal agencies, 75% of US hospitals, over 8000 pharmacies, 3400 dialysis centers, and more.

The eHealth Exchange formally adopted and added the *Image Exchange Use Case* requirements to their approved *use cases* in November 2016, but to date, this use case has not yet been implemented by Participants to exchange ra diology images.

## Conclusion

While each of these organizations operates differently from each other, they each play an integral role in interoperability. The barriers facing seamless and efficient health information exchange are complex and layered, yet each organization is working towards a common goal of improving patient care through easy health information exchange, including image exchange. It is expected, with the evolution of the DICOMweb, IHE, and HL7 FHIR [12] standards gaining momentum, there will be various opportunities to enable innovations leading to streamlined workflows for all stakeholders including providers and patients.
